# Research on the Influence of Tool Surface Texture on Cutting Performance Based on Finite Element Method

**DOI:** 10.3390/mi13071091

**Published:** 2022-07-10

**Authors:** Shujing Wu, Dazhong Wang, Jiahui Yin

**Affiliations:** School of Chemistry and Chemical Engineering, Shanghai University of Engineering Science, Shanghai 201620, China; wdzh168@126.com (D.W.); m010216125@sues.edu.cn (J.Y.)

**Keywords:** tool, AISI1045, finite element method (FEM), cutting performance

## Abstract

As research progresses, the surface texture tool can significantly reduce the cutting heat and cutting force. However, the tool surface texture width, depth, and spacing also have an impact on the cutting performance. Using the Taguchi method and finite element analysis, the changing laws of cutting temperature, pressure, stress distribution, and cutting force were studied. The results showed that the tool texture width had the greatest influence on the cutting performance, followed by the tool texture depth and spacing. The increase of tool texture width lead to the decrease of cutting temperature, stress distribution, and cutting force, while the effect of texture depth on cutting stress distribution was more significant. Cutting performance could be improved by optimizing the texture size and structure of the cutting tool. This research has theoretical significance for improving the cutting performance of cutting tools.

## 1. Introduction

The study of tool surface texture has been gaining popularity in recent years. Although the dry cutting process can reduce the pollution and waste of the cutting fluid, the dry cutting process also tends to increase the friction and adhesion between the tool and the chip contact area [1,2,3,4,5]. More researchers have begun to change the contact area between the tool and the workpiece to reduce tool wear and friction. Tool surface texture constitutes a new research direction.

Sugihara et al. conducted various experiments and simulation studies to demonstrate the cutting performance of tool textures. They studied the effect of different tool texture surfaces on tool life. By milling the workpiece section, the flank texture developed in this research can significantly reduce friction. They also conducted various cutting experiments to research the tool pit texture, which brought about a significant improvement on the tribological properties of the tool rake face and a reduced speed of crater wear [6,7]. Compared with tool groove texture, it has a superior performance in the wet cutting process. Some experimental studies have shown [8] that the high-speed cutting of Inconel 718 nickel-based alloys by a cubic-boron-nitride tool with additional texture on the flank surface can not only improve surface quality of the workpiece and cutting performance, but also increase the life of the service tool. Functional microstructure surfaces exhibit excellent performance in many interfacial contact fields. For improving the accuracy of texture topography prediction, the profile of cutting edge was considered into the simulation model according to coordinate transformation. The UVAM experiments were carried out on difficult-to-machine materials (Ti6AL4V) in order to verify the accuracy of numerical design, surface simulation, and texture generation. The relationship between the machining quality and the ultrasonic milling parameters were also revealed.

In addition, the wettability of surface textures was observed. The results indicated that the micro textures generated by UVAM contributed to the interface modification. In summary, the research on the texture generation and interference in UVAM can expand the scope of application in the field of interface contact.

For the purpose of improving the workpiece’s machining quality, the tool is surface textured [9]. The tungsten carbide tool is used for turning aluminum alloys. The side of the tool is machined with parallel and vertical grains. The researchers used ABAQUS to analyze the chips during cutting. The formation mechanism was simulated and studied. The boundary conditions were set as placement and rotation with a velocity of 0.2 m/s, as well as a workpiece meshing rate of 0.0005. The research results showed an increased tool life and a significantly improved surface quality in comparison with ordinary tools. In the cutting process, the tool was concerned with the tribological properties between the tool and the workpiece. If the tool wore sharply, then the cutting efficiency and the workpiece’s surface quality would be reduced, and the tool’s service life will be shortened. In this regard [10], the rake face of the tool was processed with triangular micro/nano texture by laser and fluorinated by fluorosilane. The tribological properties of the tool’s rake face were evaluated through simulation experiments, friction experiments, and wear experiments. 

Traditional wet cutting fluids are harmful to operator’s physical and mental health, as well as to the environment. While researchers are constantly exploring auxiliary processing methods, such as minimum quantity lubricant (MQL), they are equally concerned about how to improve traditional dry cutting. Roughness is one of the important indicators for judging the quality of surface processing. In order to efficiently predict the surface roughness of nickel metal during milling, a multivariate signal monitoring system for nickel metal processing status was built and used with the sound level meter, the three-way vibration sensor, the industrial camera, and the roughness measuring instrument to collect noise, vibration, surface texture, and roughness during milling [11]. The results showed that the prediction accuracy of the obtained roughness model based on PSO-LSSVM was 92.54%. The research could provide a theory guide for scientific monitoring of the surface roughness of milling workpieces. The tribological properties of dry hard cutting could be improved by texturing the tool surface; Kishor [12] designed six different surface textures and conducted dry cutting experiments. The results showed that the textured tool could replace wet cutting [13]. Reference [14] used cemented carbide tools to dry cut alumina ceramics, and the use of flank textured tools could reduce tool wear. A textured tool was used to cut AISI 1045 without the use of cutting fluid, and workpieces cut by micro-textured tool would cause secondary cutting, which would increase cutting friction, cutting force, hardness, and chip deformation, etc. [15].

In order to determine the effect of tool micro-texture and magnetic nanofluid coupling on the tribological properties of the tool, a laser was used to engrave micro-scale grooves parallel to the main cutting edge on the rake face of the tool, and a comparative study was carried out with ordinary tools. The cutting fluid was selected to contain 30% magnetic nanofluid, and it was compared and analyzed with ordinary cutting fluid. The results of turning experiments demonstrated that coupling tool micro-texture with magnetic nanofluid may lower the cutting force, enhance the surface roughness, and reduce the tool wear, while, moreover, the turning effect was better than that of the traditional turning [16]. Antifriction performance of the tool was improved by using a composite lyophilic texture tool surface, and the polycrystalline diamond tool was used to turn the Ti6Al4V titanium alloy in condition with minimal lubrication. As per the law of wear and tear, the results revealed that polycrystalline diamond tools with lyophilic texture had less cutting force, friction coefficient, and tool wear [17]. Su et al. [18] investigated polycrystalline diamond textured tools’ cutting performance in dry cutting conditions and concluded that the textured cutting tools outperformed conventional tools in terms of tribological performance. Rodrigo L. et al. [19] studied the interaction law between chips and rake face texture through experiments and showed that the interaction would affect the formation of chip morphology.

The performance of micro-textured (grooves, convexes, dimples) drills with different geometries in deep holes in Inconel 718 superalloy was studied by the finite element method. The influence of each parameter on the machining performance of the microdrill was tested by adjusting the spindle speed. A simulation model was created to examine the changing laws of thrust, temperature, and tool wear. The secondary cutting phenomenon of micro-texture was proposed, and the influence rule of the width of micro-texture on the secondary cutting phenomenon was revealed, indicating that the micro-texture could reduce the thrust of drilling [20]. Vasumathy et al. [21] conducted tool texture research, which revealed that the texture improved chip adhesion on the rake face and lowered the cutting force when compared to traditional tools. Roshan Sasi [22] studied surface textured tools, and discovered that HSS textured tools could improve cutting performance significantly. The tool surface texture was beneficial in order to improve the tribological characteristics of cutting, which was a promising cutting technology. The surface texture was usually on the rake face, and its geometric size, geometric shape, and other designs were different. Surface texture could improve tribological properties, help reduce cutting forces, and improve workpiece surface quality [13].

The research showed that the tool micro-texture had superior cutting performance, but its discussion was still focused on general simulation and general experiments; as such, its cutting performance and laws need to be further studied. This paper uses simulation and the Taguchi experiment to discuss the impact of various tool surface texture sizes on tool cutting performance by changing the width, depth, spacing, and other characteristics of the tool texture. As a result, this study will fill the gap, demonstrating both the theoretical and practical significance.

## 2. Numerical Simulation

### 2.1. Workpiece (AISI1045)

The material (AISI1045) properties for the workpiece can be found in Table 1.

We choose the Johnson-Cooke’s model to describe the material’s plastic properties. Table 2 displays the parameters. The constitutive equation of the Johnson’s Cooke model is defined in Equation (1) [24].

(1) $$\sigma =\left(A+B{\bar{\varepsilon }}^{n}\right)\left(1+C\ln\left(\frac{\frac{.}{\varepsilon }}{\frac{.}{\varepsilon_{0}}}\right)\right)\left(1-{\left(\frac{T-T_{room}}{T_{melt}-T_{room}}\right)}^{m}\right)$$

where σ is the flow stress *A* is the yield strength, *B* is the stiffness modulus, ε is strain, $\dot{\bar{\varepsilon }}$ is the strain rate, ${\dot{\bar{\varepsilon }}}_{0}$ is the reference strain rate, *T* is the temperature of the workpiece, *T_room_* is the room temperature, and *T_melt_* is the melting temperature.

### 2.2. Tool (Cubic-Boron-Nitride)

The cubic-boron-nitride is synthesized from hexagonal boron-nitride and catalyst at extremely high pressures and temperatures. The cubic-boron-nitride is a high-tech product after a synthetic diamond is invented. It has a high hardness, thermal stability, chemical inertness, good appearance, and excellent properties, such as a wide band gap width. Its hardness is second only to diamond, but its thermal stability is significantly greater than that of diamond, and the iron series metal elements have a greater chemical stability. The grinding performance of cubic-boron-nitride grinding tools is excellent, not only for the processing of difficult grinding materials, but also to effectively improve the productivity and grinding quality of the workpiece. The adoption of cubic-boron-nitride has made a significant contribution to metal processing, resulting in a major shift in grinding and a second leap in grinding technology.

### 2.3. Parameters of Tool Texture

We used circular texture to research the cutting performance. Meanwhile, we used the Taguchi method for a comparative analysis of the simulation. The Taguchi method was an experimental method which was put forward by Taguchi in the 1990s. Its main analysis tool was the table of the orthogonal and ratio of the signal-to-noise.

**Figure 1:**
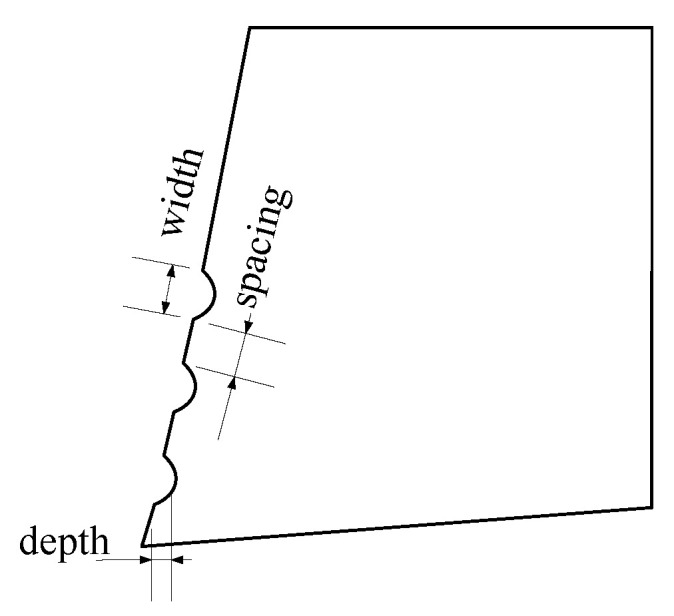
The surface texture tool.

In this paper, we used the orthogonal array, which includes the three factors as well as the three levels. The texture’s width, depth, and spacing were the influencing factors for our research. Figure 1 displays the definition of width, depth, and spacing. There are three levels in the factor, and Table 3 assumed the parameters setting. Table 4 shows the orthogonal array.

### 2.4. Metal Cutting Deformation

**Figure 2:**
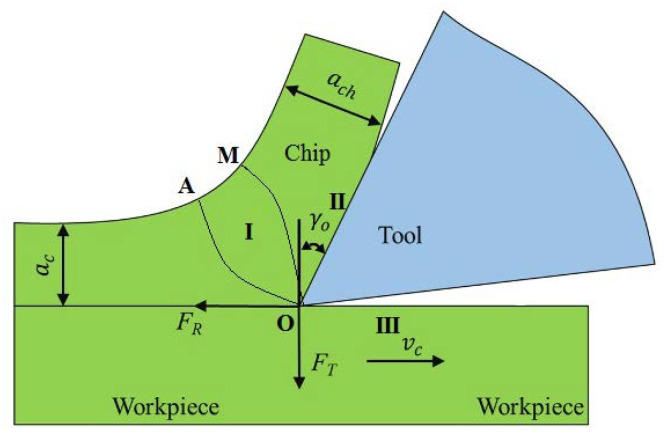
Distribution of metal cutting deformation areas.

During the metal cutting process, there were three different deformation areas in the cutting area, as shown in Figure 2.

(1) The first deformation area occurred between the OA and the OM in the cutting layer (see Figure 2). Plastic deformation and shear slip appeared in this area.
(2) The second deformation area refers to the area where the chip extends out and into contact with the rake face of the tool when it extends out of contact with tool and friction occurred.
(3) The third deformation area refers to the area where the cutting edge and the tool’s back deformed the machined surface layer near the cutting edge. The three deformed areas had their own characteristics and there are mutual connections and mutual influences.

## 3. Discussion

### 3.1. The Effect of Temperature

**Figure 3:**
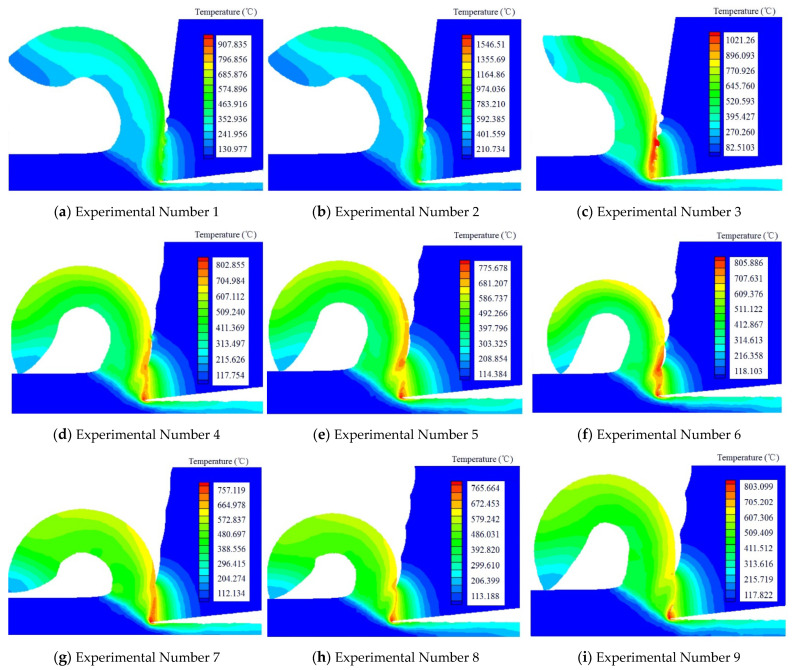
The cutting temperature produced by different texture tools.

We research the cutting performance of textured tools from two aspects: the temperature distribution in the cutting process, and the radius of the curvature of the chip (see Figure 3).

(1) We found that when the nine kinds of texture tools cut the workpiece, the third type of texture had the highest temperature, while the seventh type of texture had the lowest temperature. By comparing the parameters of the third type of texture and the seventh type of texture, we found that the space between the two textures was the same, but the temperature was quite different, ranging from 1020 °C to 750 °C. Therefore, the space between textures had little influence on temperature.
(2) We found that the first type of texture had the smallest radius of curvature, while the sixth type of texture had the largest radius of curvature. By comparing the data of the two textures, the space between the two textures was found to be the same. Therefore, the space between textures had little effect on the radius of curvature during the chip formation.

**Figure 4:**
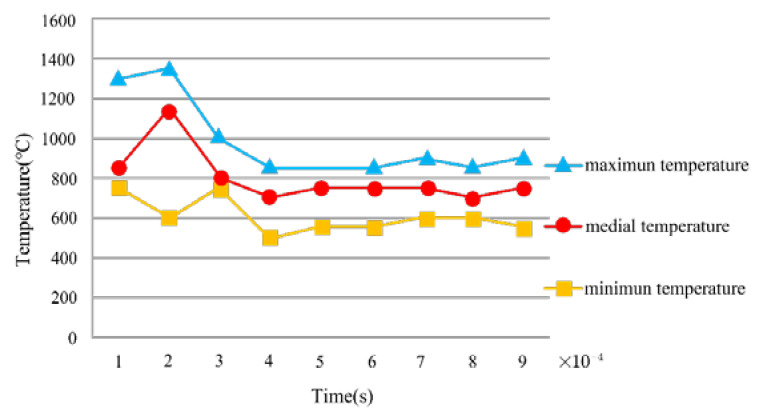
The regulation of the temperature variation.

In addition, as Figure 4 showed, we also studied the cutting performance by comparing the horizontal and the longitudinal data of the tool temperature. We found that when the texture width was 60 μm, the temperature rose significantly as the depth increased. However, when the texture width was 80 μm and 100 μm, the temperature started to become unsteady during the increase of the depth. In both cases, the cutting temperature was the lowest when the depth was 15 μm, but the change was not obvious. In contrast, at the same depth, the temperature dropped significantly as the width increased. Therefore, it was discovered that the width of the texture tool had a great effect on the temperature by analyzing the simulation data.

In conclusion, the width, depth, and spacing of texture cutting tools had varying effects on the cutting heat in the cutting process. The width of the texture had the biggest effect on temperature, whereas the spacing of the textures had the least influence on temperature. As such, while cutting hard-working metal materials, the width of the texture must be carefully chosen to reduce the heat of cutting.

### 3.2. The Effect of Pressure

**Figure 5:**
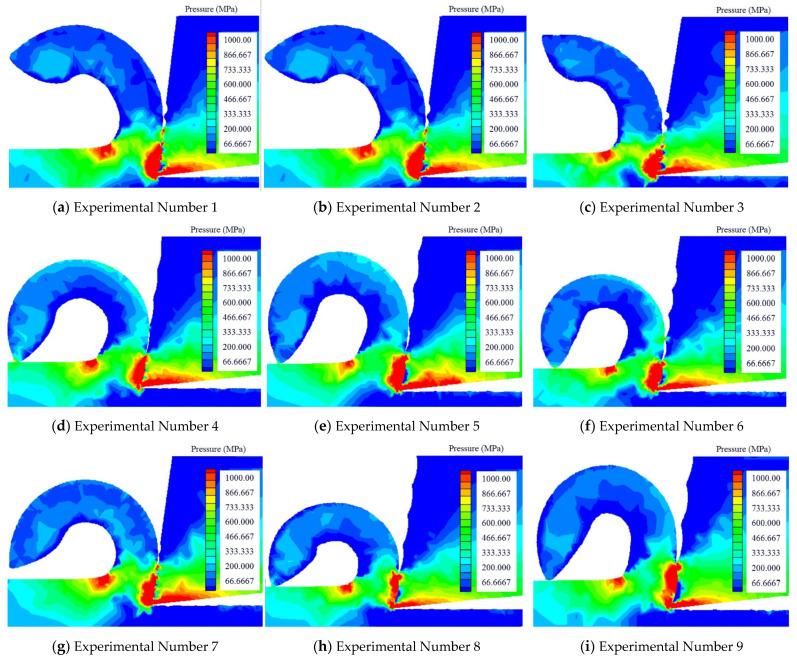
The cutting temperature produced by different texture tools and the radius of curvature of the chip.

From Figure 5, we can see that most of the pressures in the cutting process were concentrated on the tool nose, the flank face, and the second deformation zone. We studied the changes of the pressure area from three aspects.

(1) We considered the width as the standard. We divided the nine groups of experiments into three categories with the same width. When the texture width was the same, the depth and spacing increased correspondingly. Wei found that the pressure area of that flank face decreased. However, with the increase of the depth and spacing, the pressure area of the second deformation zone began to increase. Therefore, the increase of depth and spacing had a corresponding effect on the pressure in the cutting process.
(2) We used depth as the standard. We divided the nine sets of experiments into three categories with the same depth. When the depth of texture was the same, the width and spacing increased correspondingly. We found that the overall pressure area in the cutting process decreased. When the width and spacing of texture increased from 80 μm to 100 μm, the stress area decreased significantly.
(3) We divided the nine sets of experiments into three categories according to the standard of spacing. When the spacing was the same, the width of texture increased, and the depth of texture changed unknowingly, but the total pressure area decreased. In the whole process, when the width of texture reached 100 μm, the stress area in the cutting process was the smallest. We found that the change of width had great influence on the change. When the texture width was 100 μm, the pressure in the cutting process was the smallest. Secondly, the change of texture depth could reduce the pressure area. Finally, the spacing of texture had little effect on reducing the pressure.

### 3.3. The Effect of von Mises Stress

**Figure 6:**
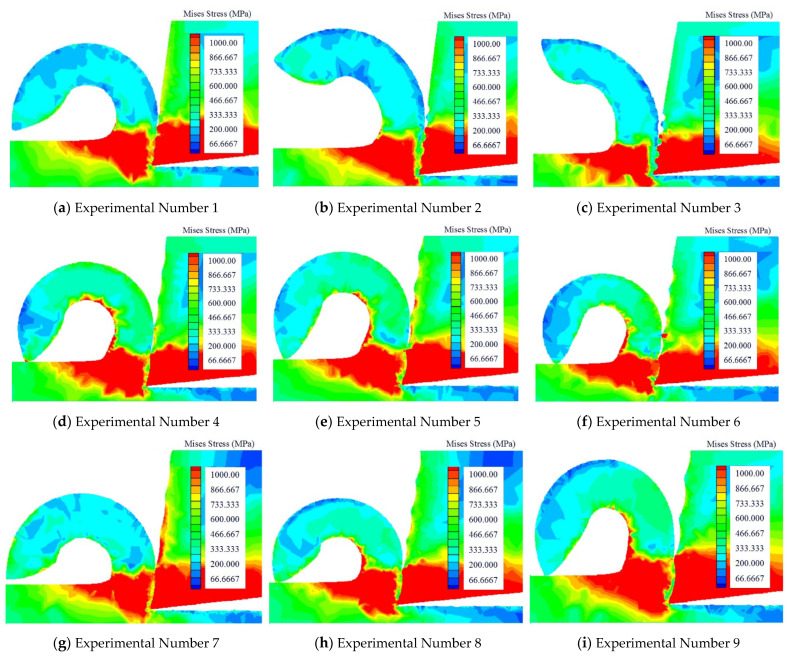
The cutting stress produced by different texture tool.

The stress distribution during the cutting process is defined in Figure 6.

(1) Under the condition that the width of the texture was the same, the depth of the texture and spacing would change. The research results showed that, during the cutting process, the stress in the tool-chip contact area reduced as the tool stress was reduced.
(2) Under the condition that the depth of the texture was the same, the width of the texture and spacing would change. The research results showed that the tool stress and chip contact area changed very slightly.
(3) Under the condition that the spacing of the texture was the same, the width of the texture and depth would change. The research results showed that the overall force decreased as the width increased, and the force condition over the whole cutting process may have changed as the depth increased.

Summarizing the above three cases, it could be found that the depth of texture change had the biggest influence on the stress distribution, followed by width of the texture, while the distance between the textures had the least effect on the stress distribution.

### 3.4. The Change of Cutting Force

This research used the Taguchi method to examine the influence of grain size on the cutting force in the metal cutting process. The quality characteristics of signal-noise ratio that the Taguchi method proposed included large-scale characteristics, expected characteristics, as well as small characteristics. By optimizing the texture size parameters, the resulting cutting force was minimized. The minimum characteristic calculation formula is shown in Equation (2) [26]:

(2) $$\frac{S}{N}=-10\log\frac{1}{n}\left(\sum_{i=1}^{n}y_{i}^{2}\right)$$

where n is total number of measurements and $y_{i}$ is the measurement.

**Figure 7:**
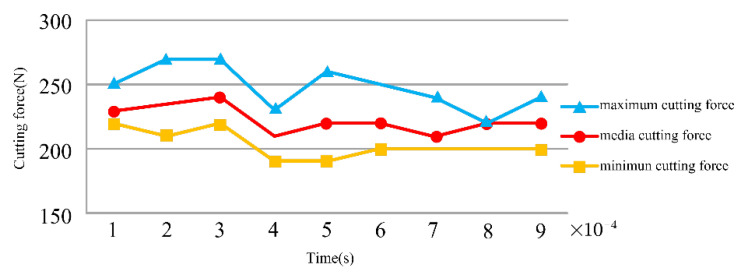
The change of cutting force.

Analyzing Table 5 and Figure 7, the magnitude of the cutting force was relevant to the width of the surface texture, depth, and spacing during the cutting process. According to Table 6, the cutting force was most affected by the width of the surface texture and was least affected by the depth of the surface texture.

## 4. Conclusions

This paper examined the influence of the law of tool texture size on cutting performance. Below is the summary.

(1) During the cutting process, the width of the texture had the biggest influence on the cutting temperature. When the texture width reached approximately 100 microns, the cutting temperature was lowest, the texture’s depth had the second highest influence on the cutting temperature, and the spacing of the texture had the least impact on the cutting temperature, which could often be ignored. Some high-hardness materials would create a lot of cutting heat during the cutting process, and the choice of width of the texture was crucial.
(2) Regarding the research on the cutting pressure distribution, the biggest influencing factor of pressure distribution was the width of the texture, followed by its depth, and, finally, the texture spacing had the smallest impact on the cutting pressure. When the chips were difficult to cut off, a lot of pressure was generated during the cutting process, and it had a strong link with the width of the texture.
(3) The influence of the texture depth on stress distribution was the largest, while the influence of texture width on stress distribution was second, and the influence of texture spacing on stress distribution was the smallest. Due to the change in the chip contact area, the cutting stress distribution would also change. When the depth of the texture increased, the contact area of the chips could be reduced.
(4) The research of cutting forces in the metal cutting process showed that the surface texture’s width had the greatest influence on the cutting force, followed by the spacing and depth of the texture. If the strength of the workpiece material and hardness were higher, the deformation resistance and cutting force would be greater. If a material of similar strength and hardness had greater plasticity and toughness, the cutting force increased as the cutting deformation increased. The cold deformation was strengthened, and the friction ecoefficiency rose. Therefore, when the cutting force was large, the width of the texture must be chosen properly.

Based on the above studies, the influence of the different sizes of the surface texture and forms on the cutting performance of the tool varies greatly and is found that it also affects the surface quality of the workpiece. Therefore, to produce the optimum cutting effect, it would be necessary to select a tool with an appropriate surface texture size for cutting the workpiece.

## Figures and Tables

**Table 1:** Material properties for AISI1045 steel [23].

Material Properties	AISI 1045
Density (kgm^−3^)	7800
Poisson’s Ratio	0.3
Conductivity (Wm^−1^°C^−1^)	44.5
Inelastic Heat Fraction	0.9
Specific Heat (JKg^−1^°C^−1^)	420
Expansion Coefficient (°C^−1^)	1.2 × 10^−6^

**Table 2:** The parameters of plastic properties [25].

A (Mpa)	B (Mpa)	C	n	m
553.1	600.8	0.0134	0.234	1

**Table 3:** Experimental influence factors and parameter setting.

	Influence Factors	Level 1	Level 2	Level 3
A	Width (μm)	60	80	100
B	Depth (μm)	10	15	20
C	Space (μm)	10	20	30

**Table 4:** Orthogonal experimental design.

Experimental Number	Factor A	Factor B	Factor C
1	1	1	1
2	1	2	3
3	1	3	3
4	2	1	2
5	2	2	3
6	2	3	1
7	3	1	3
8	3	2	1
9	3	3	2

**Table 5:** Experimental results and S/N ratio.

Experimental	A	B	C	F/N	S/N
1	1	1	1	230	−47.23
2	1	2	2	240	−47.60
3	1	3	3	235	−47.42
4	2	1	2	210	−46.44
5	2	2	3	220	−46.85
6	2	3	1	220	−46.85
7	3	1	3	210	−46.44
8	3	2	1	220	−46.85
9	3	3	2	220	−46.85

**Table 6:** The S/N ratio of the force.

	A	B	C
Level 1	−47.42	−46.70	−46.98
Level 2	−46.71	−47.10	−46.96
Level 3	−46.71	−47.04	−46.90
Δ	0.71	0.4	0.08
Ranking of importance	1	2	3

## Data Availability

This study did not report any data.
